# The neuropathological diagnosis of Alzheimer’s disease

**DOI:** 10.1186/s13024-019-0333-5

**Published:** 2019-08-02

**Authors:** Michael A. DeTure, Dennis W. Dickson

**Affiliations:** Department of Neuroscience, The Mayo Clinic Florida, 4500 San Pablo Road, Jacksonville, FL 32224 USA

**Keywords:** Alzheimer’s disease, Neuropathology, Amyloid plaques, Neurofibrillary tangles

## Abstract

Alzheimer’s disease is a progressive neurodegenerative disease most often associated with memory deficits and cognitive decline, although less common clinical presentations are increasingly recognized. The cardinal pathological features of the disease have been known for more than one hundred years, and today the presence of these amyloid plaques and neurofibrillary tangles are still required for a pathological diagnosis. Alzheimer’s disease is the most common cause of dementia globally. There remain no effective treatment options for the great majority of patients, and the primary causes of the disease are unknown except in a small number of familial cases driven by genetic mutations. Confounding efforts to develop effective diagnostic tools and disease-modifying therapies is the realization that Alzheimer’s disease is a mixed proteinopathy (amyloid and tau) frequently associated with other age-related processes such as cerebrovascular disease and Lewy body disease. Defining the relationships between and interdependence of various co-pathologies remains an active area of investigation. This review outlines etiologically-linked pathologic features of Alzheimer’s disease, as well as those that are inevitable findings of uncertain significance, such as granulovacuolar degeneration and Hirano bodies. Other disease processes that are frequent, but not inevitable, are also discussed, including pathologic processes that can clinically mimic Alzheimer’s disease. These include cerebrovascular disease, Lewy body disease, TDP-43 proteinopathies and argyrophilic grain disease. The purpose of this review is to provide an overview of Alzheimer’s disease pathology, its defining pathologic substrates and the related pathologies that can affect diagnosis and treatment.

**Electronic supplementary material:**

The online version of this article (10.1186/s13024-019-0333-5) contains supplementary material, which is available to authorized users.

## Background

### Historical information

Alois Alzheimer first described the neurodegenerative disease that would bear his name more than 100 years ago, and today the cardinal features of amyloid plaques and neurofibrillary tangles that he described are still required for its pathological diagnosis [1]. Alzheimer’s disease (AD) is a progressive neurodegenerative disease most often characterized by initial memory impairment and cognitive decline that can ultimately affect behavior, speech, visuospatial orientation and the motor system, and it is the most common form of dementia [2]. Variant syndromes with early focal atrophy do not always follow this traditional presentation, and pathological subtypes of AD have been described [3]. Clinical AD dementia cannot be definitively diagnosed until post-mortem neuropathologic evaluation, though research institutes capable of assessing amyloid and tau burden in living patients are challenging this historic paradigm [4]. AD is also characterized by a long asymptomatic preclinical phase, and cognitively normal individuals can also have the disease [5]. Furthermore, AD is rarely found without other neurodegenerative co-pathologies as observed in the Mayo Clinic Brain Bank data in Table 1. It is so tightly associated with old age that there is speculation it is a normal part of aging [6]. Currently, there are no disease modifying therapies for Alzheimer’s disease [7].

**Table 1:** Comorbidities in 1153 Patients with Pathologic Diagnosis of AD. The majority of AD cases were observed to have pathologic comorbidities as observed in the Mayo Clinic Brain Bank 2007–2016. Plus sign (+) in the column on pathological diagnosis of AD indicates additional pathologies beyond the primary and secondary diagnoses listed. Bold indicates significance from the pure AD cases (Student t-Test, *p* < 0.01)

Pathological Dx	Cases (n)	Age (yr)	Brain (g)	Braak	Thal
AD	243	76.5 ± 10.8	1077 ± 159	5.5 ± 0.7	4.7 ± 0.6
AD/LBD	175	76.0 ± 10.2	1053 ± 151	5.6 ± 0.6	4.8 ± 0.6
AD/LBD+	206	**81.8 ± 9.3**	**1025 ± 170**	5.6 ± 0.6	4.8 ± 0.5
AD/Vas	113	**84.5 ± 7.9**	1070 ± 164	**5.3 ± 0.8**	4.6 ± 0.8
AD/Vas+	77	**86.0 ± 6.4**	1043 ± 142	**5.3 ± 0.7**	4.8 ± 0.6
AD/CAA	42	76.1 ± 11.6	1089 ± 163	5.6 ± 0.6	4.6 ± 0.6
AD/CAA+	110	**80.1 ± 9.0**	1086 ± 163	5.6 ± 0.6	4.7 ± 0.7
AD/HpScl	27	**86.0 ± 6.9**	1088 ± 158	5.4 ± 0.7	5.0 ± 0.0
AD/HpScl+	51	**87.8 ± 6.9**	**1012 ± 174**	5.4 ± 0.7	4.8 ± 0.6
AD/Other	59	79.3 ± 10.9	1044 ± 164	5.4 ± 0.8	4.7 ± 0.7
AD/Other+	50	81.9 ± 11.2	1019 ± 186	5.3 ± 0.8	4.5 ± 0.8

### Epidemiology

It is estimated that more than 47 million people in the world are affected by dementia, and as of 2018 the cost of these diseases was expected to surpass $1 trillion annually [8]. AD is the most common form of dementia, accounting for 60 to 80% of the cases, with less than half expected to be pure AD and the majority expected to be mixed dementias [9]. The other most common causes of dementia include vascular dementia, Lewy body dementia and Parkinson’s disease with dementia, frontotemporal lobar degeneration and normal pressure hydrocephalus with each of these accounting for between 5 and 10% of cases; and of these, vascular dementia and Lewy body dementia are most often associated with mixed pathology, including concurrent AD [9]. These debilitating and financially devastating diseases are expected to increase into the middle of the century, and it is anticipated that greater than 131 million individuals will be affected by 2050 as the population ages [8]. Aging is the strongest risk factor for AD, with the incidence for all dementias doubling every 6.3 years from 3.9 per 1000 for ages 60–90 to 104.8 per 1000 above age 90 [10]. Prevalence is estimated at 10% for individuals over 65 years and 40% for those over 80 years [2]. The exploding personal and financial costs call for effective pre-clinical diagnosis and treatments to halt disease progression before symptomatic onset.

### Etiology

Dominantly inherited familial AD (FAD) can be caused by mutations in amyloid precursor protein (*APP*), presenilin 1 (*PSEN1*) or *PSEN2* genes. These rare familial forms of AD account for less than 1% of the cases. FAD can present as early as age 20, with the average age of onset of 46.2 years [11]. Early onset Alzheimer’s disease (EOAD) is defined by those affected before age 65; and though they are slightly more common than FAD cases, they account for fewer than 5% of the pathologically diagnosed AD cases. EOAD often has an atypical presentation and an aggressive course [12]. Similarly, most Down’s syndrome patients with a partial or full chromosome 21 trisomy, which includes the region on chromosome 21 where APP resides [13], have Alzheimer type pathology by age 40 with many developing clinical symptoms after 50; the majority have dementia by age 65 [14]. More common late onset AD (LOAD) is considered sporadic, although genetic risk factors have been identified, most notably apolipoprotein E gene (*APOE*) [7]. Age, family history in a first degree relative, and *APOE4* genotype confer the greatest risks of developing AD [14]. Individuals carrying a single copy of the *APOE4* polymorphism have an odds ratio for AD of 3 compared to non-carriers. Those homozygous for *APOE4* have an odds ratio of 12 [7]. Furthermore, *APOE4* allele appears to confer risk for vascular dementia, Lewy body dementia, Down’s syndrome and traumatic brain injury [15]. Other risk factors for LOAD including TREM2, ADAM10 and PLD3 have been identified using genome wide association studies to implicate nearly 30 genes that not only affect APP and tau directly but also modulate cholesterol metabolism, endocytosis and immune response, among those with known functions [16, 17]. Understanding the role of these and newly identified risk factors should provide insight into mechanisms that drive Alzheimer’s pathogenesis.

### Pathology of Alzheimer’s disease

#### Macroscopic features

**Fig. 1:**
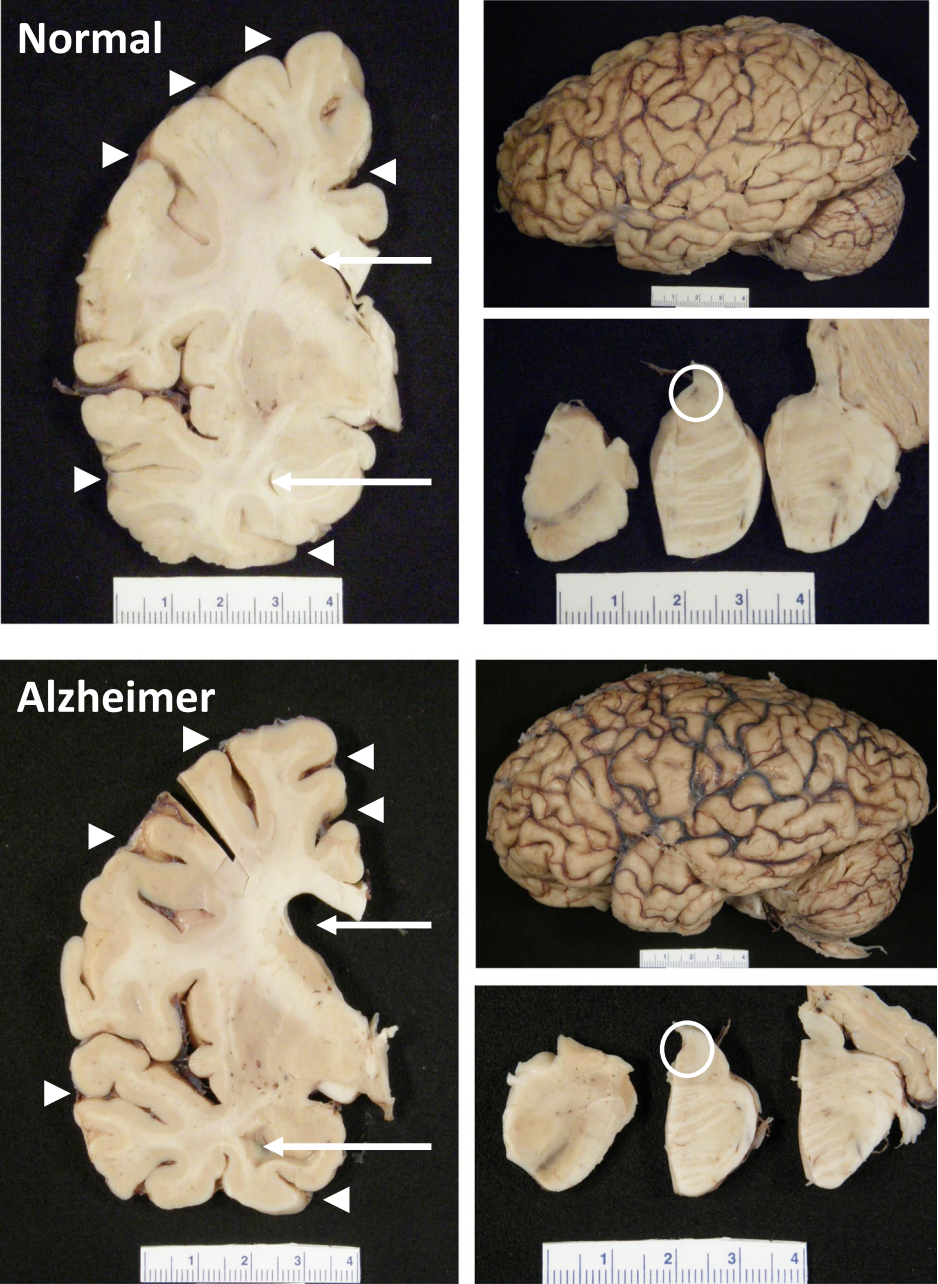
Gross Anatomy of Alzheimer’s Brain. Lateral view of an Alzheimer’s brain can show widening of sulcal spaces and narrowing of gyri compared to a normal brain. This may be more readily observed in coronal sections as indicated by the arrowheads, and this atrophy is often accompanied by enlargement of the frontal and temporal horns of the lateral ventricles as highlighted by the arrows. Additionally, loss of pigmented neurons in the locus coeruleus is commonly observed in the pontine tegmentum as shown with the open circle. None of these features is exclusive to Alzheimer’s disease

The pathologic diagnosis of AD remains the gold standard for diagnosis. While certain features of AD can be ascertained on macroscopic examination, no single feature or combination of features is specific, but certain features are highly suggestive of AD. The AD brain often has at least moderate cortical atrophy that is most marked in multimodal association cortices and limbic lobe structures. The frontal and temporal cortices often have enlarged sulcal spaces with atrophy of the gyri, while primary motor and somatosensory cortices most often appear unaffected [18]. There is increasing recognition of atrophy in posterior cortical areas in AD, most notable the precuneus and posterior cingulate gyrus, driven in part by functional imaging studies [19, 20]. As a result of this atrophy, there is often enlargement of the frontal and temporal horns of the lateral ventricles as shown in Fig. 1, and decreased brain weight is observed in most affected individuals. None of the macroscopic features are specific to AD, and unaffected clinically normal people may have moderate cortical atrophy, especially affecting frontal lobes, with volume loss mostly affecting white matter [21]. Medial temporal atrophy affecting amygdala and hippocampus, usually accompanied by temporal horn enlargement is typical of AD [18, 22, 23], but can be seen in other age-related disorders such as hippocampal sclerosis or argyrophilic grain disease. Another macroscopic feature commonly observed in AD is loss of neuromelanin pigmentation in the locus coeruleus as shown in Fig. 1 [23]. None of these observations alone are specific to AD, but often they can be highly supportive, especially in the absence of macroscopic changes specific for other neurodegenerative diseases.

#### Microscopic features

The definitive diagnosis of AD requires microscopic examination of multiple brain regions employing staining methods that can detect Alzheimer type neuropathologic change [24], with diagnosis based upon the morphology and density of lesions and their topographic distribution. Several of the brain regions that are vulnerable to Alzheimer type pathologic change are also vulnerable to other disease processes, such as α-synucleinopathy and TDP-43 proteinopathy. Mixed pathology is common. Indeed in the Mayo Clinic Brain Bank from 2007 to 2016 (Table 1**,** Additional file 1: Figure S1), the majority of AD cases had coexisting non-Alzheimer pathologies, and comorbidities increased in frequency with age. Furthermore, when the original clinical diagnoses were examined for cases with pure AD pathology, it is clear (Table 2**,** Additional file 2: Figure S2) that a number of clinical syndromes can masquerade as Alzheimer’s disease.

**Table 2:** Clinical Diagnoses of 227 Patients with a Pathologic Diagnosis of Pure AD. More than a third of pathologic AD cases on the Mayo Clinic Brain Bank from 2007 to 2016 were not expected to have AD. Plus sign (+) in the column on clinical diagnosis indicates additional clinical diagnoses are supported. Bold indicates significance differences from the pure AD cases (Student t-Test, *p* < 0.01)

Clinical Dx	Cases (n)	Age (yr)	Brain (g)	Braak	Thal
AD	109	77.3 ± 11.2	1061 ± 163	5.5 ± 0.7	4.7 ± 0.7
AD+	35	**73.0 ± 8.5**	1069 ± 138	5.6 ± 0.6	4.8 ± 0.5
LBD	16	80.9 ± 9.8	1073 ± 158	5.3 ± 0.6	4.5 ± 0.8
CBS	12	71.3 ± 7.4	1008 ± 169	5.9 ± 0.3	5.0 ± 0.0
FTD	10	**66.4 ± 8.9**	1103 ± 128	5.5 ± 1.1	4.9 ± 0.4
Aphasia	6	72.2 ± 7.1	940 ± 109	5.9 ± 0.2	4.8 ± 0.4
Other	21	78.2 ± 10.3	1132 ± 155	5.4 ± 0.8	5.0 ± 0.0
MCI	11	**87.7 ± 6.9**	1162 ± 136	**4.9 ± 0.6**	5.0 ± 0.0
Normal	7	85.9 ± 6.6	1251 ± 120	4.7 ± 0.8	3.0 ± 0.0

As first observed over 100 years ago [25], the presence of extracellular amyloid plaques and intracellular neurofibrillary tangles is required for diagnosis. Additionally tau-positive neuropil threads and dystrophic neurites can be detected, as well as activated microglia and reactive astrocytes. Additionally eosinophilic Hirano bodies, granulovacuolar degeneration (GVD) and cerebral amyloid angiopathy (CAA) are also common [18, 23]. The result of these lesions is the loss of synapses and neurons in vulnerable regions leading to the symptoms commonly associated with AD. Evidence suggests that amyloid deposition and tau pathology in AD can precede structural changes in the brain, including hippocampal volume loss and decreased glucose metabolism, by decades [26]. As the disease progresses, downstream clinical features of memory loss, social dependence and motor abnormalities eventually become manifest [5, 6]. The usual symptoms of early AD (e.g., memory deficits) can be different in some AD subtypes, and indeed, clinical features lack specificity for a pathologic process, but rather indicate brain regions or systems affected by the disease process [3, 4]. This finding is highlighted by the fact that nearly one third of patients with pathological findings of high likelihood Alzheimer’s disease [24] were thought to other disorders, and 3% were considered clinically normal at the time of their last clinical exam (Table 2).

### Amyloid plaques

**Fig. 2:**
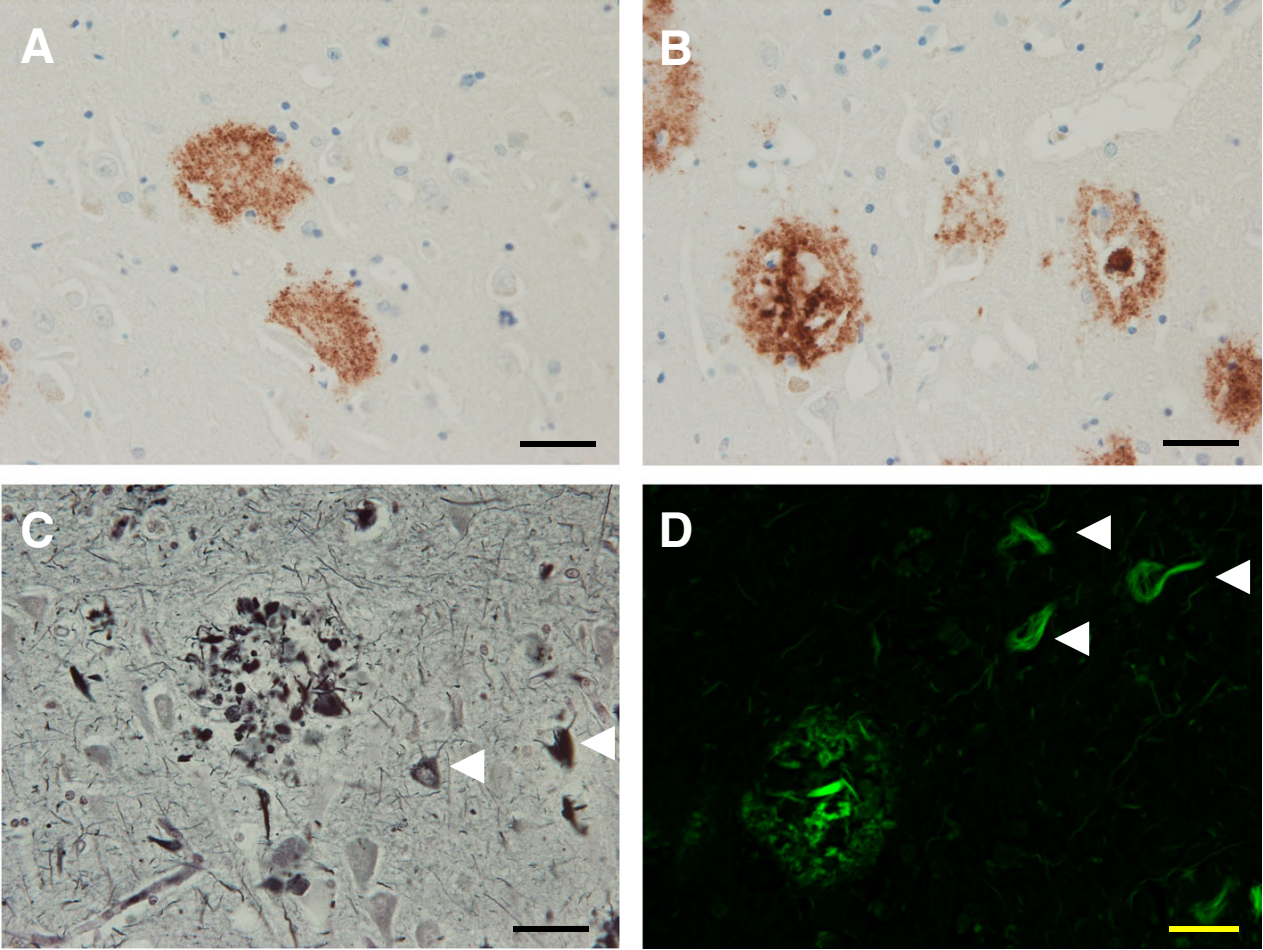
Alzheimer Senile Plaques. Immunohistochemistry of affected Alzheimer’s tissues using antibodies directed against Aβ peptides demonstrates the presence of both diffuse (**a**) and dense core (**b**) senile plaques. These dense core plaques are often associated with neuritic elements that can stain filamentous tau and correlate with disease severity. Neuritic AD plaques are readily observed using Bielchowsky silver staining (**c**) or Thioflavin S staining (**d**). These stains can also label neurofibrillary tangles as shown by the arrowheads. The scale bars are 40 μm

Senile amyloid plaques or “miliary foci” as described by Alzheimer in the description of Alzheimer’s disease and originally that same year by Oskar Fischer are formed by the extracellular nonvascular accumulation of Aβ40 and Aβ42 peptides that result from the abnormal processing of amyloid precursor protein by the β- and γ-secretases and an imbalance in the production and clearance pathways [1, 27–29]. These small 4 kDa peptides (A4) fold into a beta-pleated sheet structures that are highly fibrillogenic. The Aβ filaments bind congophilic dyes and produce birefringence upon exposure to polarizing light as is characteristic of amyloid [30]. Work using antibodies to A4 protein, now recognized as Aβ peptides, demonstrated they are involved in early formation of amyloid plaques that were not captured with congophilic dyes [31]. Additional Aβ peptides containing between 38 and 43 amino acids are also detected, but Aβ42 is the most fibrillogenic and the predominant component of amyloid plaques in AD [6]. There are many reviews describing the generation of Aβ peptides [32], and these pathways remain viable therapeutic targets. The terminology for Aβ amyloid plaques can be confusing. Nearly a dozen types of nonvascular amyloid deposits have been described, but the two types of amyloid plaques most commonly observed in AD are diffuse plaques and dense core plaques [29, 33] as shown in Fig. 2a, b [23, 29]. Diffuse plaques form initially in the neuropil and stain weakly by thioflavin S and other amyloid binding dyes (e.g., Congo red). They commonly lack argyrophilia on Bodian silver stains, and they do not show preferential accumulation of activated microglia and reactive astrocytes. In comparison, dense core plaques have dense reticular or radiating compact dense amyloid and are intensely positive with thioflavin S fluorescent microscopy and Congo red, suggesting they contain more fibrillogenic forms of Aβ [29, 34, 35]. More importantly, a subset of dense core plaques have neuritic elements as shown in Fig. 2c, d, and these cored neuritic plaques (NPs) can be associated with tau-positive or dystrophic neurites, the latter demonstrated with a variety of markers including synaptic and APP immunohistochemistry [33]. Dense cored NPs are also accompanied by synaptic loss, activated microglia and reactive astrocytes [23, 29, 36]. In contrast, diffuse plaques often lack neuritic components, though diffuse neuritic plaques can be observed in advanced AD [33]. The diffuse plaques are positive with Aβ immunohistochemistry and contain filamentous Aβ at the ultrastructural level, but it is not certain whether diffuse plaques are a part of pathological aging or an early stage in the maturation of neuritic Aβ plaques [29]. Indeed, it appears as many as 8 types of nonvascular amyloid plaque deposits can form after initial deposition of Aβ and that the morphology and type of plaques can vary from region to region [29]. Plaques composed almost exclusively of dense cores lacking neuritic components have been termed “burnt out” plaques [18]. Most importantly the neuritic plaques with dense amyloid and tau-positive neurites are believed to be closely associated with neuronal loss and cognitive decline in Alzheimer’s disease [37, 38].

### Neuritic plaques

Cored neuritic plaques containing tau-positive neurites usually have a central zone of dense amyloid, sometimes forming a compact core. The dense core can display radiating Aβ fibrils, and it is in these peripheral zones of the plaque where dystrophic neurites and activated microglia are concentrated, that lends credence to the hypothesis that Aβ drives neuronal degeneration and cognitive decline in AD [18, 39]. Neuritic plaques frequently contain activated microglia and reactive astrocytes, and their processes intermingle with neuritic elements in the plaque periphery. Some of the dystrophic neurites associated with neuritic plaques contain tau filaments, which can have a paired helical filament morphology with electron microscopy [23]. These are termed “type 1” dystrophic neurites, and it is suggested they occur in regions receiving input from neurons bearing neurofibrillary tangles in their soma [29, 36]. The dystrophic neurites in neuritic plaques are heterogeneous [36]. In addition to tau-positive neurites, some dystrophic neurites contain neurofilament proteins, suggesting that cytoskeletal changes are part of the neurodegenerative process [40]. Additionally degenerating mitochondria, lysosomal bodies and vesicles, some with ubiquitin-immunoreactivity, can accumulate in a subset of plaque-associated dystrophic neurites, indicating that trafficking and protein degradation pathways are affected [23]. More recent studies demonstrate that exogenous Aβ fibrils lead to cell death and disruption of membrane integrity in a cell culture model [41]. In fact, even the presence of dystrophic neurites, which are postulated to be benign age-related neurites, still provides evidence that amyloid plaques negatively affect the integrity neuronal processes in their vicinity [36]. While dense core neuritic plaques are thought to be more closely associated with neuronal loss in AD [38], diffuse amyloid plaques with neuritic elements are observed in advanced disease though their disease relevance and relationship to the formation of dense core neuritic plaques is not certain [33]. Understanding the links between amyloid-driven neuritic pathology, more widespread tau neuronal and thread pathology, as well as neuronal loss remains an area of active research.

### Distribution of amyloid plaques

In AD, senile plaque and neurofibrillary tangle formation tend to form neuroanatomically in stereotypic patterns, which has led to several staging schemes [42, 43]. One of the first attempts to stage amyloid plaques in AD was proposed by Heiko and Eva Braak. A three stage scheme was proposed, with the basal frontal and temporal lobes affected in Stage A, extension into the association neocortices and hippocampus in Stage B, and finally reaching primary cortices, subcortical nuclei and cerebellum in Stage C [42]. Within the cortical layers, layers III and Va are most affected, layers IV and Vb were affected less, and other layers were relatively spared [42]. More recently, the Braak plaque staging has been modified by his research associate, Dietmar Thal, and this scheme of amyloid “phases” using highly sensitive silver staining or Aβ antibodies [43] has been adopted by NIA-AA [44] and BrainNet Europe [45]. In Thal Phase 1 the neocortex is involved, expanding to the allocortex in Phase 2, subcortical nuclei, including the striatum, in Phase 3, with involvement of brainstem in Phase 4 and cerebellum in Phase 5 [43]. For practical work, phases 1–4 can be determined by amyloid deposition in the medial temporal lobe [29]. It is not unusual for neurologically normal patients to have Thal 1–3 amyloid phase as shown in Table 2.

### Cerebral amyloid Angiopathy

**Fig. 3:**
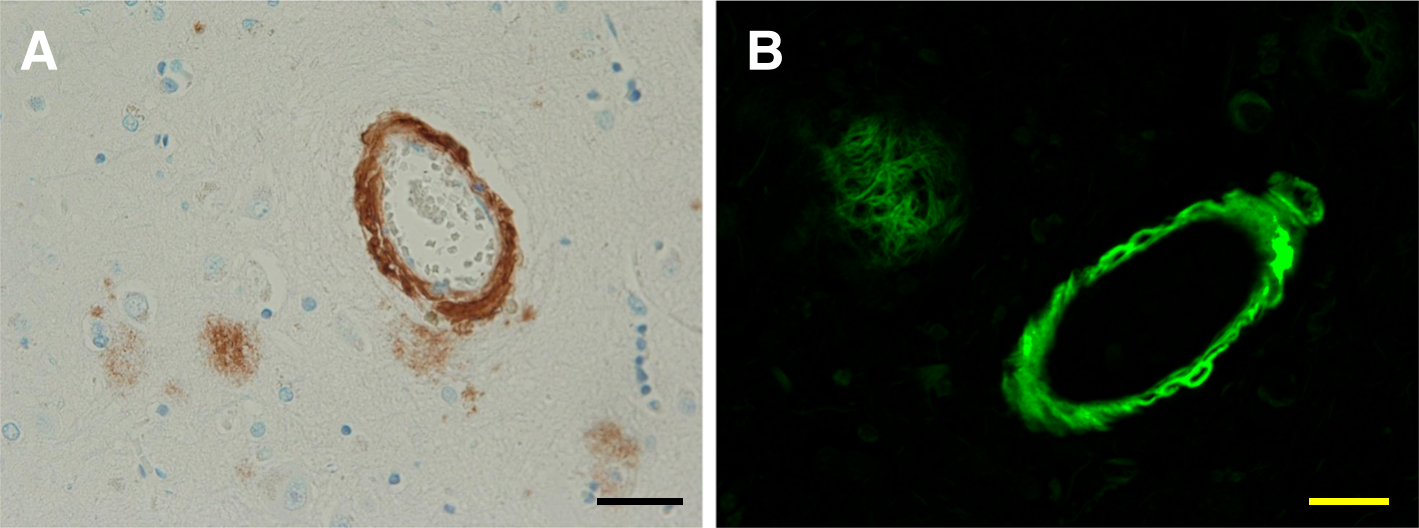
Cerebral Amyloid Angiopathy. Cerebral amyloid angiopathy or congophilic amyloid angiopathy can by visualized in frontal cortical sections using Aβ directed immunohistochemistry (**a**) or Thioflavin S staining (**b**) similar to that used to detect senile plaques, and they are believed to contribute independently to the Alzheimer’s disease course. Scale bars are 40 μm

Aβ peptides not only deposit as amyloid plaques in the brain parenchyma, but also in cerebral blood vessels. It is estimated that 85–95% of AD cases have at least some degree of cerebral amyloid angiopathy. In the Mayo Brain Bank 13% of confirmed AD cases have moderate-to-severe CAA (Table 1) that can be confirmed using Aβ immunohistochemistry or thioflavin S fluorescent microscopy Figs. 3a, b [46]. Amyloid deposits in CAA are enriched in Aβ40 (while parenchymal deposits are enriched in Aβ42 species) and can affect small arteries, arterioles and even capillaries of the gray matter of the cerebral cortices and in leptomeningeal vessels [18, 23]. In fact, two types of CAA have been described. Type 1 CAA affects capillaries, arterioles and small arteries and it is 4-times more likely to be associated with *APOE4*. Type 2 CAA affects arterioles and small arteries, but not capillaries and is 2-times more likely to associated with *APOE2* [47, 48]. Interestingly the parietal and occipital cortices are more vulnerable than the frontal and temporal lobes, and leptomeningeal arteries are more vulnerable than parenchymal vessels [23]. Severe CAA can impair blood flow and produce ischemic lesions or small infarcts, while severe CAA can lead to lobar hemorrhages typically affecting the frontal and occipital lobes [18]. The preponderance of CAA in AD and its association with an earlier age of onset support its role on the disease process, independently contributing to clinical presentations of AD [46, 49, 50]. Several methods have been proposed to score severity of CAA burden, and imaging techniques are being developed to differentiate CAA from plaque amyloid [46]. Interestingly, immunization strategies targeting Aβ peptides may be useful in alleviating amyloid plaque burden, while shifting amyloid into CAA, sometimes associated with inflammation and hemorrhage [51, 52]. The evidence from animal models suggest capillary CAA is derived from neuronal Aβ, which leads to impaired perivascular clearance, peri-capillary Aβ deposits and ultimately CAA [53]. It is postulated that the *APOE4* risk associated with capillary CAA results from less efficient transendothelial clearance of Aβ-apolipoprotein complexes compared to complexes in individuals with *APOE2* [48]. Interestingly, the severity of perivascular neuritic tau pathology has been found to correlate with perivascular Aβ accumulation, implicating amyloid deposition in driving dystrophic neurites [54].

### Neurofibrillary tangles and neuropil threads

**Fig. 4:**
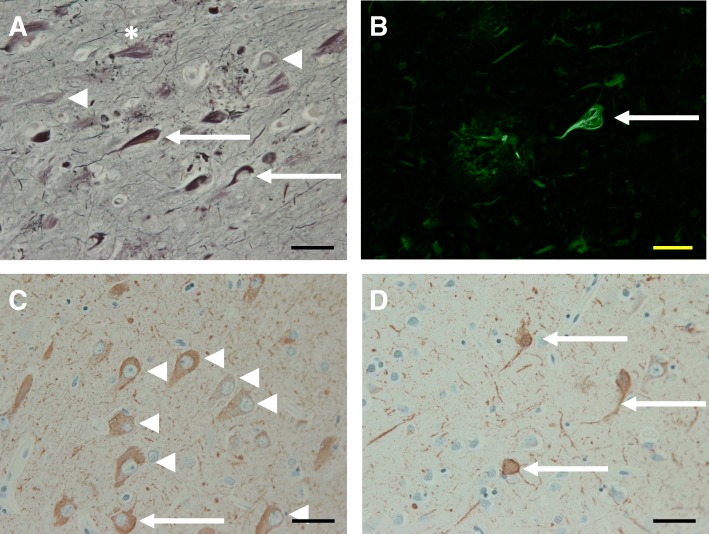
Neurofibrillary Tangles. Neurofibrillary tangles develop from intracellular pre-tangles containing misfolded tau and small tau aggregates to mature NFTs containing bundles of cross-linked tau filaments before the neuron dies and an extracellular ghost tangle (asterisk) remains. Silver staining (**a**) and Thioflavin S (**b**) capture many mature tangles (arrows) and some pre-tangles (arrowheads) along with amyloid plaques and tau neuropil threads. Development of NFTS from the pre-tangles is more easily visualized using tau immunohistochemistry (**c**, **d**). This allows the mis-localized somal tau to be distinguished readily from the bundles of PHFs in NFTS in addition to the neuropil threads that can also be pronounced (**d**). The scale bars are 40 μm

**Fig. 5:**
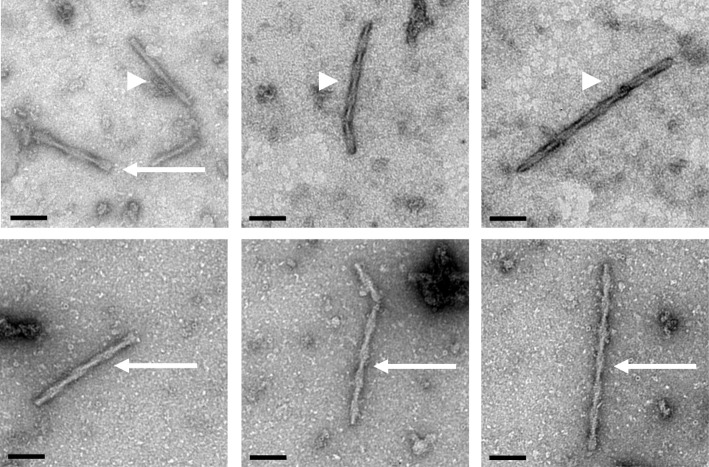
Paired Helical Filaments and Straight Filaments. Neurofibrillary tangles are composed of insoluble tau filaments that can be extracted using detergents or acids and visualized using electron microscopy and negative staining such as 2% uranyl acetate here. The paired helical filaments (arrows) and straight filaments (arrowheads) both appear to be composed of pairs of 10 nm filaments that wrap around one another with the PHFs exhibiting wider 10–20 nm modulations in diameter and the SFs narrower 10–15 nm modulations. Length bars are 100 nm

Neurofibrillary tangles (NFTs) were first described in Alzheimer’s seminal paper as “neurofibrils,” which formed thick bundles near the cell surface of affected neurons [25]. These were visible using Bielschowsky silver stain and associated with neuronal death and disintegration resulting in what are now called extracellular or “ghost tangles” as shown in Fig. 4a [1]. A neuropathologic diagnosis of AD requires both amyloid plaques, especially cored neuritic plaques, and neurofibrillary tangles composed of filamentous tau proteins. There is evidence to suggest that the latter lesions correlate better with cognitive impairment than amyloid deposits [55]. The tau filaments in AD have been termed “paired helical filaments” (PHFs) [56], as they exhibit marked periodicity when viewed with electron microscopy, and they appear to be composed of two smaller filaments of about 10 nm diameter that twist around one another forming periodic structures with a crossover distance of 65–80 nm. As shown in Fig. 5, these filaments exhibit modulations in width from roughly 10–20 nm, and their uniformity has allowed for the structural folding of the core peptide to be resolved at 3.5 A using cryoelectron microscopy [57]. Additionally, straight filaments (SFs) are observed in AD, but these exhibit less periodicity with a longer crossover distance and modulations in width from 10 to 15 nm [58]. Alzheimer straight helical filaments are different from straight half filaments that are uniformly 10 nm in diameter and observed in other tauopathies and filaments generated in vitro using recombinant tau. The composition of PHF in AD includes all 6 isoforms of tau protein, including isoforms with 3-repeats (3R tau) and 4-repeats (4R tau) in the microtubule binding domain. The repeat domain forms the core of PHF [59]. Tau proteins in AD are hyperphosphorylated and abnormally folded compared to unassembled normal tau, and they have lost their normal abilities to bind and stabilize microtubules in the axon [60]. This loss of tau function is coupled with increased aggregation properties for the abnormal tau. Both a loss of normal function and a toxic gain of function are postulated, whereby PHFs are able to co-aggregate with normal tau proteins [61]. The PHFs and SFs composed of tau protein are also observed in neuropil threads (NTs) which are dendritic and axonal elements containing filamentous tau. Neuropil threads may represent the majority of tau burden in AD, and they are thought to originate from neurons containing NFTs [62–64]. Corruption of normal tau is thought to be potentially involved in the spreading of tau pathology throughout the brain in AD.

### Distribution of neurofibrillary tangles

Detection of neurofibrillary tangles can employ traditional histological or histofluorescent staining methods (e.g., Bielschowsky silver stain or thioflavin-S) or more recently immunohistochemical techniques using antibodies against tau as shown in Fig. 4. These techniques also mark numerous neuropil threads that are believed to be part of the neuronal degeneration associated with neurofibrillary tangle formation [23]. Neurofibrillary tangles occur in three stages beginning as “pretangles” containing abnormal conformers of tau (but not polymerized into microscopic aggregates) in the cell body and dendrites of neurons. These mature into aggregated filaments in the perikarya and proximal cell processes. They can appear as “flame shaped tangles” in pyramidal neurons of the hippocampus and layer V of the association cortices and as “globose tangles” as in the basal nucleus of Meynert, raphe nuclei, substantia nigra and locus coeruleus among other places. The morphology of the tangle is driven by the type of neuron in which it forms [18]. The mature tangles displace the nucleus and other vital cellular components, and eventually the neurons die. The insoluble filaments are left in the extracellular spaces, where they associate with microglia, astrocytes and extracellular proteins (e.g., Aβ) as a “ghost tangle.” It is believed that neuronal tau pathology, leading to tangles and neuropil threads, is linked to neuronal death and cognitive decline in AD. Studies have shown that their number and location correlates with neuronal loss, disease severity and clinical course [65, 66]. The relationship of neurofibrillary tangles to cell death is not understood, though mutations in the tau gene (*MAPT*) on chromosome 17 lead to tau accumulation and neuronal loss in other neurodegenerative diseases. These disorders are termed frontotemporal dementia with Parkinsonism linked to chromosome 17 [67]. Whether these correlations are related to the loss of tau function and microtubule integrity associated with these diseases or both is not resolved. Whether tangles disrupt protein homeostasis or sequester necessary cellular components is still controversial. Similar to plaque accumulation in AD, tangles typically follow a stereotypic progression.

The most widely used staging scheme for tangles was proposed in 1991 by Heiko and Eva Braak [68]. The staging scheme is widely used in neuropathologic criteria for AD [69, 70]. The earliest stages of NFT deposition in Braak Stage I are associated with tangles in the transentorhinal cortex as it transitions between the allocortical entorhinal cortex and the temporal isocortex. Here in the transentorhinal cortex as the superficial entorhinal Pre-α layer deepens in position, the multipolar neurons from the entorhinal cortex transform to pyramidal cells resembling those in the neighboring temporal isocortex. It is these Pre-α projection neurons of the transentorhinal cortex that accumulate the first NFTs in Alzheimer’s disease along with the occasional tangles observed in the CA1 sector of the hippocampus and select nuclei in the basal forebrain and thalamus [68, 71]. In Stage II, NFTs observed in Stage I become more robust and the Pre-α layer of the entorhinal cortex becomes involved along with stronger representation in the anterodorsal nucleus of the thalamus. These first two stages of NFT and NT formation are termed the “transentorhinal stages”, and NFTs are sparse in the hippocampus proper and virtually absent in the isocortex. In Stage III, Pre-α layers of the transentorhinal and entorhinal cortices are fully involved and ghost tangles begin to appear. NFTs can also be observed in the Pri-α and Pre-β layers, along with the CA1 sector and pyramidal neurons of the subiculum. Tangles in the magnocellular nuclei of the basal forebrain and anterodorsal nucleus of the thalamus also become more pronounced. In Stage IV, NFTs continue to accumulate in the CA1 region of the hippocampus and also become apparent in CA4. Additionally neuritic plaques may be observed in the corticomedial complex along with NFTs and NTs in the basolateral nuclei of the amygdala and some portions of the putamen and accumbens nucleus. All previous pathology becomes more robust during Stages 3–4 termed the “limbic stages,” and isocortical pathology remains limited with primary motor and sensory cortices markedly untouched. In Stage V the Pre-β and Pre-γ layers and most of the hippocampus are now affected, and NTs and ghost tangles become more pronounced in previously affected areas and NPs can be seen in CA1. The largest changes involved in Stage V regard the extensive involvement of the isocortex. In mild cases this may be more apparent in the basal portion of the medial frontal and the inferior portions of the temporal and occipital lobes followed by areas of the insula and the orbitofrontal cortex. In more severe cases most of the association cortices are affected, including NPs in layer III of the temporal lobes, though still only occasional NPs are observed in layer III of the primary motor and sensory cortices. In Stage VI previously affected areas continue to deteriorate and now all association areas are severely affected. Changes in the primary sensory cortex are defined by numerous NTs and occasional NFTs in layer V while the primary motor cortex pathology is still largely confined to layer III NPs. The anteroventral and reticular nuclei of the thalamus are more involved as are portions of the hypothalamus, striatum and substantia nigra. Together with Stage V these represent the “isocortical stages.” Recent modifications of the Braak scheme also consider possible earlier involvement of subcortical nuclei (e.g., locus ceruleus) [68, 72].

### Granulovacuolar degeneration

**Fig. 6:**
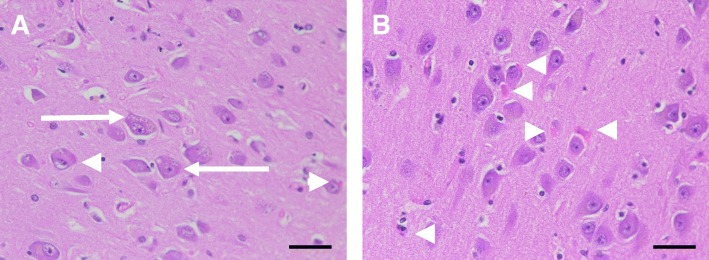
Granulovacuolar Degeneration and Hirano Bodies. Granulovacuolar degeneration is commonly observed in the neurons of AD (**a**). As indicated by the arrows, these neurons contain numerous vacuoles housing a dense granule. Additionally, Hirano bodies are often observed as eosinophilic pink rods within the neurons, as shown with the arrowheads (**b**). The scale bars are 40 μm

Additional pathologic changes are inevitably detected in AD, but not as well characterized in terms of clinical relevance. Granulovacuolar degeneration is one such finding. In addition to AD, it is detected in other neurodegenerative diseases and in some normal elderly individuals albeit in low density. These intraneuronal vacuoles were first described by Alzheimer and colleagues in 1911 are thought to be an integral component of AD [73–75]. They are 3–5 μm vacuoles with a central 0.5–1.5 μm dense granule as shown in Fig. 6a. They are labeled with lysosomal markers (e.g., acid phosphatase histochemistry) and are considered to be autophagic granules [76, 77]. They are most frequent in the cytoplasm of pyramidal neurons in the hippocampus. They are double membrane bound vacuoles at the EM level, and they have immunoreactivity for cytoskeletal elements, including neurofilament and tau, in addition to the lysosomal markers (e.g., cathepsin D and LAMP1). There is some debate as to their significance and origin, as they also contain epitopes related to apoptosis and stress granules [74, 77, 78]. Microscopically, these lesions can be viewed by Bielschowsky’s silver stain, hematoxylin and eosin staining, though they are not positive with thioflavin S fluorescent microscopy or Congo red stains [74]. Staging suggests these structures accumulate in the hippocampus first, followed by the entorhinal cortex and temporal neocortex and finally the amygdala, thalamus, cingulate gyrus and even association cortices [79]. As these vacuoles contain relatively few epitopes associated with pathological tau, it is not certain if they represent a cellular defense or are more intimately involved in AD pathogenesis [74]. Most recently, phospho-ubiquitin mitophagy markers label granulovacuolar bodies and their abundance has been shown to correlate with neurofibrillary tangle density, suggesting they may be a cellular response to damage in the neurons [80].

### Hirano bodies

Hirano bodies were first described in Parkinson’s disease complex of Guam patients as eosinophilic intracytoplasmic inclusion bodies found in the presence of NFTs and GVDs, but not amyloid plaques [76, 81]. These rod-like inclusions shown in Fig. 6b are located in neuronal dendrites and are rich in F-actin and actin binding proteins, and they can be seen with hematoxylin and eosin staining. They have been reported in middle age and elderly normal individuals, as well as a number of disorders in addition to AD [81]. They are most commonly observed in the CA1 region of the hippocampus. They are more frequent and numerous in AD than in normal controls [82]. Their role in AD pathogenesis remains unclear [23]. More recently, animal models expressing mutant actin binding proteins suggest Hirano bodies are associated with impaired synaptic responses and decreased spatial working memory [83]. More research needs to be done in order to better understand their pathogenesis and role in Alzheimer’s disease.

### Inflammatory response

Microglial cells are phagocytes that operate within the brain, monitoring their territories for pathogen exposure or deteriorating neurons, which promotes their migration to the site and subsequent activation and at times antigen presentation [84, 85]. Normally they play a role in synaptic monitoring and turnover, but under conditions of stress or deterioration (e.g., protein aggregates such as Aβ amyloid fibrils and tau paired helical filaments) they become activated and are observed around senile plaques, and their numbers increase in promotion to neuronal damage associated with NFTs and NT [86, 87]. Microglial activation is characterized as an innate immune response, which can be activated by multiple factors in the local environment. Receptors on microglia can bind Aβ fibrils, driving an inflammatory response similar to the M1 (proinflammatory) phenotype observed outside the central nervous system [84]. Activated ameboid microglia are frequently observed juxtaposed to amyloid deposits in plaques. This may explain the risk association of *TREM2* with AD, as this microglial and astrocytic receptor is thought to mediate microglial phagocytosis [84]. Reactive astrocytes represent the other inflammatory response observed in the brains of AD patients, and they are thought to function in the neuroprotection of damaged neurons and maintaining homeostasis. Astrocytes normally function to provide trophic support for the neurons and synapses directly connecting these structures to the critical blood supply and nutrients. In AD, they also are often observed around senile plaques, though in lower abundance compared to microglia, and at a greater distance from the plaque epicenter. It is thought they react to the cytokines and other agents produced by pro-inflammatory M1 microglia, and these changes in the astrocytes are thought to be neurotoxic [88, 89]. Reactive astrocyte burden occurs later in AD when dementia develops, and is considered to be correlate with tau burden [90]. It should also be noted that neurons and oligodendrocytes are involved in regulation of glia [85].

### Synaptic loss

Neuronal loss parallels the distribution of neurofibrillary tangles in AD, and it is a better correlate of cognitive deficits than the tau burden [91]. Still neurons with tangles can be long lasting, perhaps persisting as ghost tangles for decades [23]. Perhaps more importantly, it appears that synaptic loss precedes neuronal loss, and these effects are probably driven by amyloid and tau pathology [92, 93]. Numerous studies of synaptic protein markers and as well as electron microscopy have documented synaptic loss in AD and amnestic mild cognitive impairment that is thought to precede AD [92]. Synaptic loss appears to be the strongest correlate of cognitive decline in AD surpassing the associations with neuronal loss and tau burden [94]. Cognitive decline and decreases in verbal fluency early in AD are believed to reflect these decreases in synaptic density in the hippocampus and medial temporal lobes. One of the earliest studies demonstrated that declines in synapses are likely the result of axonal dysfunction affecting the presynaptic termini [95–97]. Gene expression studies in AD validate these findings, as proteins involved in synaptic vesicle trafficking and neurotransmitter recycling, in addition to structural elements of the synapse, are affected [92]. These pathogenic changes appear to be directly related to AD pathology, and their effects on synaptic function are associated with clinical symptoms. The route of synaptic damage may be multifocal, as impaired axonal transport resulting from tau dysfunction may be complemented by the arrival of hyperphosphorylated tau in dendritic spines and concomitant impaired synaptic transmission [98]. Interestingly, it is noted the remaining synapses become larger and more robust, lending credence to a compensatory synaptic hypothesis in AD [23].

### Alzheimer’s diagnoses

#### Subtypes of AD

Atypical clinical presentations have been reported in patients that are ultimately determined to have AD at autopsy and have been recognized for almost 50 years [99]. Indeed there was speculation for a time as to whether true variants existed or whether clinical heterogeneity resulted from examination at different points in the disease course [100]. Often these are thought to be clinical misdiagnoses (Table 2), and many have comorbidities that complicate clinical diagnoses. Three major subtypes of AD can be classified by the relative density of hippocampal neurofibrillary tangles with respect to neocortical tangles [3]. Two atypical AD subtypes are defined in this manner: “hippocampal sparing AD” and “limbic predominant AD.” Hippocampal sparring AD often has an earlier age of onset than typical AD and a more rapid rate of cognitive decline. In contrast, limbic predominant AD often has a later age of onset life and a more slowly progressive cognitive decline. Interestingly, 30% of the hippocampal sparing cases may have an atypical clinical presentation. These cases can have increased cortical atrophy that may be focal or asymmetric, which often leads to clinical symptoms other than defects in episodic memory [101]. Deficits in language and semantic knowledge, executive functions and visuospatial deficits are observed in AD, but they are not usually the clinical presentation [101]. Patients with primary progressive aphasias presenting as agrammatic, semantic or logopenic variants most commonly have frontotemporal lobar degeneration, but hippocampal sparing AD can also present in this manner [7, 102]. Another atypical clinical presentation sometimes associated with hippocampal sparing AD is posterior cortical atrophy (PCA). In addition to AD, corticobasal degeneration and prion disease can cause PCA [103]. Progressive executive dysfunction is most often associated with frontotemporal lobar degeneration, but AD, especially hippocampal-sparing AD, can have this presentation [104]. Genetic analyses of *APOE* suggest *APOE4* is more closely linked to typical or limbic predominant than hippocampal sparing AD [3]. Amyloid plaque pathology is increased in cases with capillary CAA. Capillary CAA is also linked to *APOE4* and a variant (C766T (rs1799986)) in the LDL receptor related protein 1 (LRP-1), a receptor for apolipoproteins, which suggests that capillary CAA my also represent a subtype of AD [105, 106]. Data shown in Table 3 and Additional file 3: Figure S3 from the Mayo Clinic Brain Bank illustrates the frequency of primary AD presenting with non-amnestic syndromes. In the future, use of antemortem biomarkers for tau and amyloid, including amyloid or tau imaging, may improve recognition of atypical presentations of AD [107].

**Table 3:** Pathologic Diagnoses in 626 Patients with Clinical Diagnosis of AD. The majority of clinical AD cases as observed in the Mayo Clinic Brain Bank from 2007 to 2016 were found to have co-pathologies. Plus sign (+) in the column on pathological diagnosis of AD indicates additional pathologies beyond the primary and secondary diagnoses listed. Secondary AD indicates a primary pathological diagnosis other than Alzheimer’s disease though AD changes are noted and contributing. Bold indicates significance differences from the pure AD cases (Student t-Test, *p* < 0.01)

Path Dx	Cases (n)	Age (yr)	Brain (g)	Braak	Thal
AD	108	77.3 ± 11.2	1060 ± 166	5.6 ± 0.7	4.7 ± 0.7
AD/LBD	73	79.2 ± 9.7	1017 ± 122	5.7 ± 0.5	4.9 ± 0.5
AD/LBD+	104	**83.1 ± 8.3**	**981 ± 168**	5.6 ± 0.6	4.8 ± 0.6
AD/Vas	41	**84.4 ± 5.0**	1007 ± 148	5.4 ± 0.8	4.6 ± 0.7
AD/Vas+	46	**86.9 ± 6.6**	1052 ± 135	**5.2 ± 0.6**	4.8 ± 0.5
AD/CAA	17	75.3 ± 14.8	1019 ± 146	5.8 ± 0.4	4.8 ± 0.4
AD/CAA+	53	80.3 ± 9.1	1045 ± 156	5.6 ± 0.5	4.7 ± 0.8
AD/HpScl	20	**86.7 ± 5.8**	1104 ± 143	5.5 ± 0.6	5.0 ± 0.0
AD/HpScl+	24	**87.3 ± 7.5**	**977 ± 181**	5.7 ± 0.6	5.0 ± 0.0
AD/Other	21	80.9 ± 11.0	1032 ± 135	5.5 ± 0.6	4.8 ± 0.6
AD/Other+	25	81.8 ± 10.6	**950 ± 176**	5.4 ± 0.7	4.6 ± 0.7
Secondary AD	15	79.5 ± 8.1	1075 ± 167	**4.6 ± 0.9**	4.4 ± 0.9
No AD	74	**83.0 ± 7.7**	1049 ± 167	**2.9 ± 1.3**	**1.5 ± 1.7**
Normal	5	87.6 ± 4.5	1140 ± 158	2.9 ± 0.2	1.4 ± 1.3

#### Neuropathologic criteria

The pathologic diagnosis of AD recognizes that any amount of Alzheimer neuropathologic change is abnormal. The most current set of criteria for the neuropathological assessment of Alzheimer’s disease termed the 2012 National Institute on Aging-Alzheimer’s Association Guidelines are based on the semi-quantitative measure of Thal Aβ amyloid phase, Braak NFT stage and CERAD neuritic plaque score, and they are applicable to patients with or without dementia [69]. This contrasts to the original set of criteria produced in 1985 by the National Institute on Aging, which focused on age-related amyloid plaque density (with silver stains or thioflavin S fluorescent microscopy) without regard to type of plaque [108]. These were subsequently revised in 1991 by the Consortium to Establish a Registry for Alzheimer’s Disease (CERAD), which focused on neuritic plaques in the frontal, temporal and parietal cortices and included clinical presence of dementia to determine if AD was the definite, probable or possible cause of the observed symptoms [109]. Neither of these early criteria included a measure of neurofibrillary tau burden, and subsequently in 1997 the National Institute on Aging and Reagan Institute combined the CERAD neuritic plaque scoring and Braak staging to determine if clinical dementia had a high, intermediate or low probability of being caused by AD [110]. These CERAD and NIA-RI criteria also considered the effects of vascular and Lewy body disease [4]. Ultimately these criteria were revised in the 2012 NIA-AA Guidelines as it became more universally acknowledged that AD pathology can be present in the absence of clinical symptoms though there are updated clinical guidelines available for ascribing dementia to AD pathology before death [111]. The current criteria use an ABC scoring system that requires the presence of amyloid plaques and tau neurofibrillary tangles to describe the amount of AD neuropathological change ranging from none to low to intermediate to high amounts of change. The A score is measured by grouped Thal phase, the B by Braak stage and the C by neuritic plaque CERAD score. These guidelines include two measures of amyloid plaque burden as their independent value was not known [69]. To reach thresholds for intermediate and high Alzheimer’s disease neuropathologic change, cases can have low Thal phase, but they must have a Braak stage greater than III and a CERAD score in the moderate to frequent range. Again this system retains adjustments for age and considers Lewy body disease, vascular disease and hippocampal sclerosis among other comorbidities. Additionally a practical assessment companion article was published at the same time describing the brain sections and staining techniques useful in deriving these scores [24]. Neuropathologic criteria align with revised clinical framework for considering Alzheimer type neuropathologic change in living individuals using antemortem biomarkers to assess amyloid (A), tau (T) and neurodegeneration (N). With some combinations associated with AD preclinical stage (A+, T+, N-), symptomatic stages (A+, T+, N+) or non-AD disease processes (A-, T ±, N+) [112].

### Comorbidities in Alzheimer’s disease

#### Cerebrovascular pathologies

Vascular dementia is most often sporadic and related to a range of cerebrovascular lesions determined by the types of vessels involved and the location of the ensuing brain damage [113]. It represents a heterogeneous group of disease processes that are often observed together and increase in frequency with age. It represents the most common concurrent pathology in AD [18, 113, 114]. In the Mayo Clinic Brain Bank approximately 16% of the cases with AD also have significant cerebrovascular pathology, and patients with mixed pathology were significantly older than pure AD. The most commonly observed forms of vascular dementia are multi-infarct dementia (MID), strategic infarct dementia and subcortical vascular encephalopathy [115], and these are often caused by atherosclerosis, small vessel disease (SVD), and CAA [113, 114, 116]. Atherosclerosis is observed in larger meningocerebral arteries, as well as the internal carotid and vertebral arteries and arteries of the circle of Willis. These fibrofatty intimal lesions and can lead to infarcts (macroscopic and microscopic in nature) or hemorrhages [113–115]. MID can be caused by atherosclerosis, SVD or CAA, but atherosclerosis-related thrombosis and emboli are a major cause of MID [115]. Similarly, SVD caused by arteriolosclerosis and cardio-embolic disease can produce cortical and subcortical microinfarcts, which correlate with cognitive deficits in vascular dementia [113]. Cerebral penetrating and lenticulostriate arteries can be damaged, resulting in cortical infarcts, lacunar infarcts, microinfarcts and white matter lesions, which drive both strategic infarct dementia and subcortical vascular encephalopathy (previously referred to as Binswanger’s disease) [113, 115]. In strategic infarct dementia damage to the hippocampus or thalamus can be caused by SVD or embolic events though capillary CAA occlusion has been observed in these regions [115]. SVD subtypes include arteriosclerosis, lipohyalinosis and arteriolosclerosis and commonly affects arteries in the basal ganglia, white matter and brainstem [116]. CAA can affect leptomeningeal and intracerebral arteries leading to cortical microinfarcts, microhemorrhages, lacunar infarcts and white matter lesions [113]. In the later stages of CAA, the cerebellum, basal ganglia and thalamus are affected, with white matter changes occurring last [115]. Capillary CAA has been associated with microinfarcts in CA1 of the hippocampus and can contribute to hippocampal neuronal loss that is not associated with TDP-43 pathology or epilepsy [117]. Most importantly, it appears that cerebrovascular pathology acts synergistically with AD in driving cognitive impairment and dementia, and that it is highly dependent on the type of cerebrovascular disease and the location of lesions [118]. Certainly as lifespans increase, the frequency of cerebrovascular pathology can be expected to increase and its contributions to age-related cognitive deficits need to be considered.

### Lewy related pathology

Lewy body dementia is a term that encompasses dementia with Lewy bodies (DLB) and Parkinson disease dementia (PDD) [119]. Lewy related pathology is associated with pathologic deposition of α-synuclein in neuronal cell bodies as Lewy bodies and neuronal cell processes (mostly axons) as Lewy neurites. Specific neuronal populations in the central and peripheral nervous systems are vulnerable to Lewy-related pathology. In Lewy body dementia, Lewy related pathology affects corticolimbic regions and is often associated with concurrent Alzheimer type pathologic change [120, 121]. In addition Lewy related pathology has been reported to be frequent in the substantia nigra of AD, and for a time it was suspected that patients with PD and coincident dementia had mixed (AD-PD) pathology [18, 23]. Now it is accepted that patients with Lewy pathology can have dementia caused strictly by the Lewy pathology, and this can be observed in patients who exhibit Parkinsonian clinical symptoms first, as in PDD, and those who develop dementia first, as in DLB. Almost a quarter of AD patients develop Parkinsonian features, but the relative contribution of substantia nigra tangles and Lewy bodies to this presentation is unresolved [122]. Lewy bodies in AD do not follow the same pattern of selective vulnerability associated with PD. In particular, Lewy pathology in AD is most frequent in olfactory bulb and the amygdala [23, 123]. The clinical significance of olfactory and limbic Lewy related pathology is uncertain. In the Mayo Clinic Brain Bank approximately 33% of the cases with AD also have Lewy related pathology as a secondary finding, and work is being carried out to determine what factors contribute to comorbidity. Evidence suggests that Aβ can directly affect α-synuclein toxicity, and it is also known that tau protein and α-synuclein can directly interact promoting their co-assembly [124–126]. Moreover there are several candidate genetic risk factors that overlap for AD and PD, most notably *APOE* and *MAPT* [127].

### TDP-43 pathology

TDP-43 protein deposition is often encountered in hippocampal sclerosis and AD [128] in addition to the frontotemporal dementias [129]. Hippocampal sclerosis of aging is now recognized to almost always include TDP-43 deposits and sometimes arteriolosclerosis that lead to intracellular and neuritic deposits of TDP-43 in the hippocampus during the preclinical state and eventually hippocampal sclerosis and cerebral atrophy in the disease state [130, 131]. This independent disease process may affectt up to 10% of clinical AD cases over age 85 without pathologically confirmed AD [130]. Typically when observed in AD, TDP-43 inclusions are first observed in the amygdala and later the hippocampus (“limbic”), followed by involvement of neocortex and subcortical areas (“diffuse”) analogous to terms used to describe distribution of Lewy-related pathology [130, 132]. Data from multiple reports suggest that between 19 and 75% of AD have TDP-43 neuronal inclusions [132]. AD patients with TDP-43 pathology tend to be older [130]. As the distribution of TDP-43 increases from limbic regions to other parts of the brain, it is associated with worsening cognition and with medial temporal atrophy on antemortem brain imaging [130, 132, 133]. TDP-43 deposits are more common in typical AD and limbic-predominant AD than hippocampal-sparing AD [134]. Similar to the increased frequency of Lewy related pathology in AD, TDP-43 is more frequent in the presence of pure AD or mixed AD/LBD compared to controls [135]. This is a rapidly expanding field of research, and more work will be needed to elucidate how TDP-43 pathology is related mechanistically to AD pathology and their downstream effects.

### Argyrophilic grain pathology

In Alzheimer’s disease, tau is routinely deposited as PHF composed of 3R and 4R tau, but in many primary tauopathies it can also be deposited as either 3R tau or 4R tau. Argyrophilic grain disease (AGD) is a 4R-tauopathy [136] with grain like deposits in neuronal dendrites, accompanied by oligodendroglial inclusions (“coiled bodies”), ramified astrocytes and ballooned neurons in the amygdala, hippocampus and medial temporal lobe [137, 138]. AGD is associated with amnestic cognitive impairment, increases in frequency with age and has been reported in up to a quarter of AD. Data from the Mayo Clinic Brain Bank supports these findings [139]. A pathologic diagnosis of argyrophilic grain disease can be confirmed with antibodies specific for 4R tau that readily distinguish grains from the tangles and threads of AD. Argyrophilic grain disease is not only more frequently observed in Alzheimer’s disease than the general population, but it also lowers the pathological threshold for the presentation of clinical dementia [140].

### Age related tau pathologies

Primary age-related tauopathy (PART) and aging-related tau astrogliopathy (ARTAG) are independent tau pathologies commonly observed in patients over 80 years of age, and PART may account for a proportion of the misdiagnosed clinical AD cases in the elderly [130, 141, 142]. Neurofibrillary pathology in PART is similar to AD, being composed of 3R and 4R isoforms. Lesions are found in the hippocampus and adjacent regions similar to those observed in Braak I-IV, but PART is distinct from AD in that it lacks amyloid plaques, and it has distinct morbidity and age range [130, 142]. The frequency of PART increases in the 80s and 90s when AD prevalence is level or declining [130]. NFTs in PART may pass through similar phases as those observed in AD, with progression from pretangles to NFTs and eventually extracellular ghost tangles. The latter, when frequent, are associated with memory complaints observed in the elderly [130, 142]. Differentiating PART from AD remains a challenge [130]. ARTAG represents astrocytic tau pathology distinct from that observed in primary tauopathies including PSP, CBD and GGT. It is associated with 4R tau deposits in subpial or perivascular thorn-shaped astrocytes or granular fuzzy astrocytes in gray matter [130]. It is recommended that location, region and severity of ARTAG be noted, but its relationship to neurological symptoms is not well characterized [130].

### Preclinical Alzheimer’s disease

Alzheimer’s pathology can be found in cognitively normal patients at the time of autopsy, and this preclinical phase can also be observed in patients with mild cognitive impairment that do not meet the criteria for clinical AD [4, 23]. At this point, it is not certain whether clinically normal individuals with AD pathology would have developed clinical manifestations had they lived longer [55, 143]. Recommendations for defining the preclinical stages of AD are designed to help improve early and accurate clinical diagnoses for potential therapeutic intervention, and as such these efforts require recognition of amyloid and tau pathology, as well as synaptic loss as substrates for the clinical manifestations of AD [144]. Those with amnestic mild cognitive impairment do not always have AD at autopsy even though they have a reported risk for developing dementia of 10–15%, and sometimes they do not have any discernable pathology at autopsy [145, 146]. Indeed criteria for the clinical diagnosis of AD take care to document dementia and progression while ruling out other likely diseases, with additional emphasis placed on patients with positive amyloid and tau biomarkers [111]. Currently, most of this work is still in the research phase. The majority of practicing clinicians lack access to amyloid and tau biomarkers. Spinal fluid assessments are not routine, and molecular imaging is currently not a reimbursed expense. The presence of amyloid plaques and tau neurofibrillary tangles defines AD, but even with the best imaging, neuropathological diagnoses will remain the standard to identify comorbidities and attribute clinical symptoms to their root cause.

## Conclusions

Alzheimer’s disease is an expanding medical crisis taking an enormous personal and financial toll on those affected and their families. The problem is exacerbated by the lack of routine diagnostic tools for identifying patients early enough in their course for treatment. Of equal concern is the lack of effective therapeutic options once the disease process is recognized. Future research is needed to determine the root causes of AD, as well as its variability in clinical presentation. Increasing awareness of comorbidities that contribute to clinical outcomes will be needed to develop the most effective treatments. Given the multi-proteinopathies associated with aging brains, eventual therapies will be multifaceted targeting the disease through multiple avenues to inhibit both primary disease pathogenesis and additional untoward cellular responses. Effectiveness of therapeutic approaches requires concurrent progress in developing specific and sensitive diagnostic tools. These challenges are considerable as are the costs to the millions of patients affected by this disease.

## Additional files

Additional file 1: **Figure S1.** Comorbidities in 1153 Patients with Pathologic Diagnosis of AD. The majority of AD cases were observed to have pathologic comorbidities as observed in the Mayo Clinic Brain Bank 2007–2016. (PPTX 52 kb)

Additional file 2: **Figure S2.** Clinical Diagnoses of 227 Patients with a Pathologic Diagnosis of Pure AD. More than a third of pathologic AD cases on the Mayo Clinic Brain Bank from 2007 to 2016 were not expected to have AD. (PPTX 52 kb)

Additional file 3: **Figure S3.** Pathologic Diagnoses in 626 Patients with Clinical Diagnosis of AD. The majority of clinical AD cases as observed in the Mayo Clinic Brain Bank from 2007 to 2016 were found to have co-pathologies. (PPTX 63 kb)

## Data Availability

All data generated during this study are included in this published article**.**

## Abbreviations

AD: Alzheimer’s Disease
AGD: Argyrophilic Grain Disease
APOE: Apolipoprotein E
APP: Amyloid Precursor Protein
ARTAG: Aging-related Tau Astrogliopathy
CAA: Cerebral Amyloid Angiopathy
DLB: Dementia with Lewy Bodies
EOAD: Early Onset Alzheimer’s disease
FAD: Familial Alzheimer’s
GVD: Granulovacuolar Degeneration
LOAD: Late Onset Alzheimer’s disease
MID: Multi-infarct Dementia
NFT: Neurofibrillary Tangle
NT: Neuropil Thread
PART: Primary Age-related Tauopathy
PCA: Posterior Cortical Atrophy
PDD: Parkinson’s Disease Dementia
PHF: Paired Helical Filament
SF: Straight Filament
SVD: Small Vessel Disease
