# Supplementary material for: The neuropathological diagnosis of Alzheimer’s disease
Source: Mol Neurodegener. 2019 Aug 2;14:32. doi: 10.1186/s13024-019-0333-5 (PMC6679484; doi:10.1186/s13024-019-0333-5)
Supplement: Supplementary file 1 — Figure S1. Comorbidities in 1153 Patients with Pathologic Diagnosis of AD. The majority of AD cases were observed to have pathologic comorbidities as observed in the Mayo Clinic Brain Bank 2007–2016. (PPTX 52 kb) [file 13024_2019_333_MOESM1_ESM.pptx]

## Slide 1
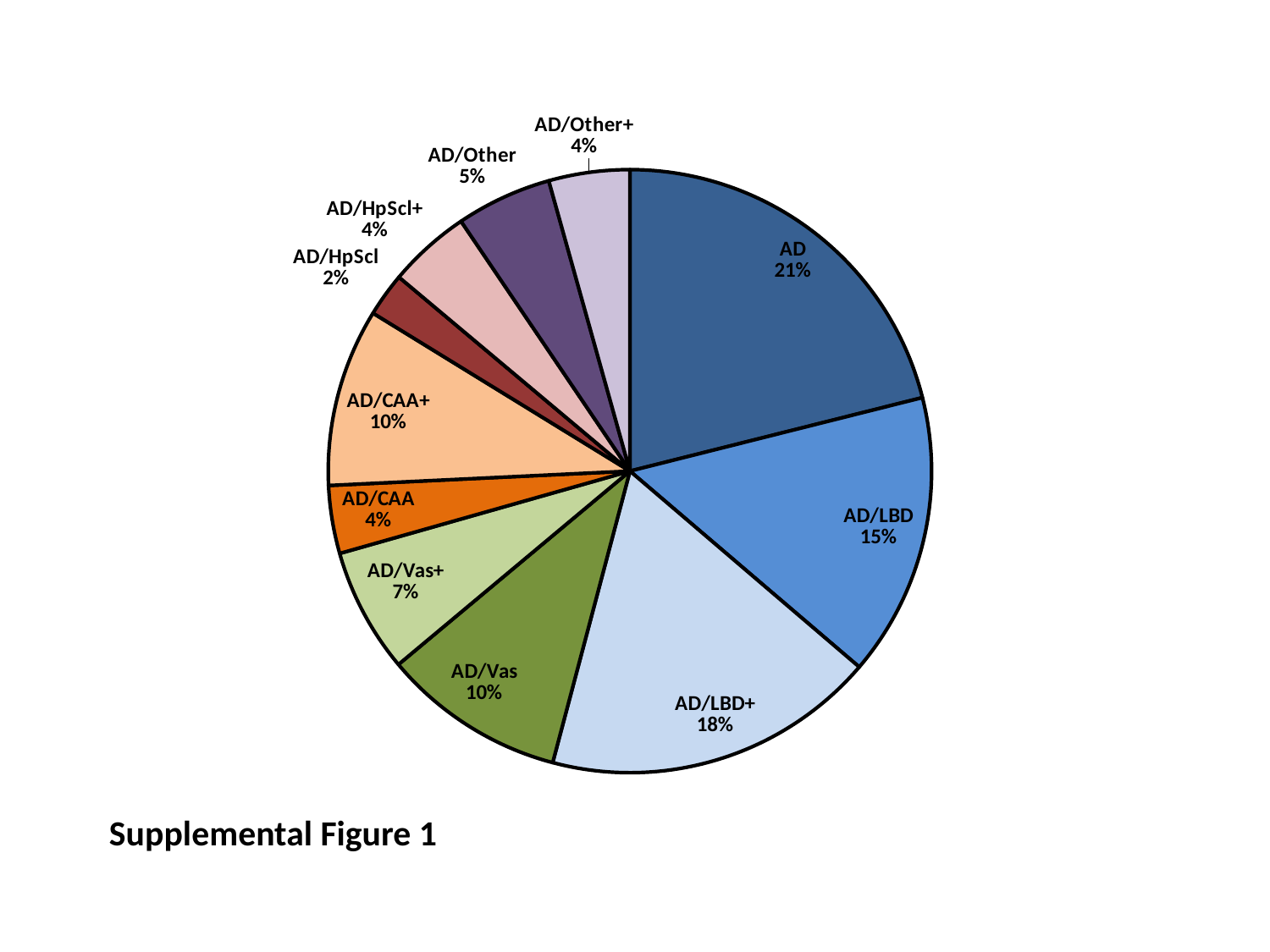

### Chart
| Category | |
|---|---|
| AD | 243.0 |
| AD/LBD | 175.0 |
| AD/LBD+ | 206.0 |
| AD/Vas | 113.0 |
| AD/Vas+ | 77.0 |
| AD/CAA | 42.0 |
| AD/CAA+ | 110.0 |
| AD/HpScl | 27.0 |
| AD/HpScl+ | 51.0 |
| AD/Other | 59.0 |
| AD/Other+ | 50.0 |Supplemental Figure 1
