# Supplementary figures and images for: The neuropathological diagnosis of Alzheimer’s disease
Source: Mol Neurodegener. 2019 Aug 2;14:32. doi: 10.1186/s13024-019-0333-5 (PMC6679484; doi:10.1186/s13024-019-0333-5)

## Slide 1
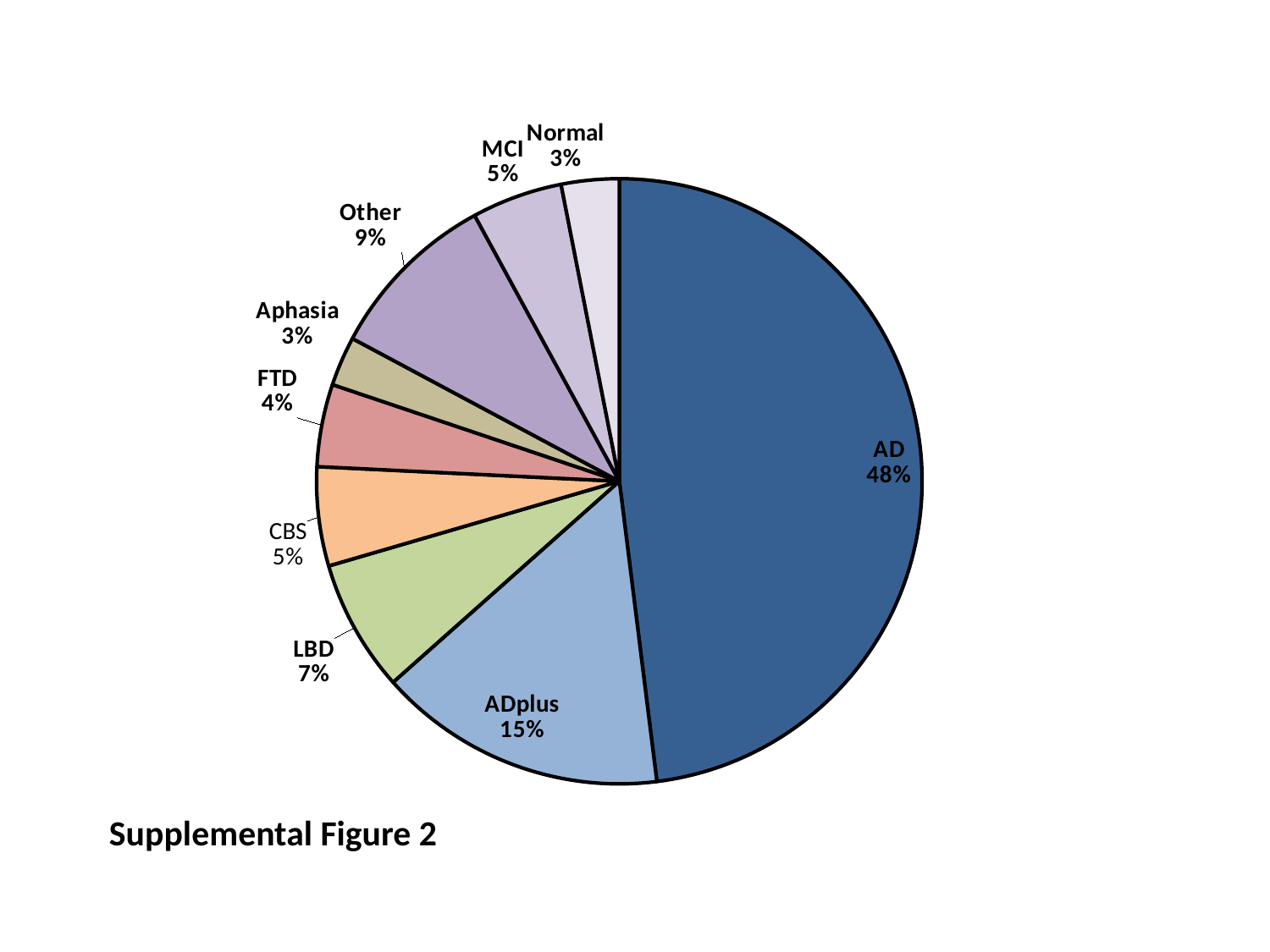

### Chart
| Category | |
|---|---|
| AD | 0.4801762114537445 |
| ADplus | 0.15418502202643172 |
| LBD | 0.07048458149779736 |
| CBD | 0.05286343612334802 |
| FTD | 0.04405286343612335 |
| Aphasia | 0.02643171806167401 |
| Other | 0.09251101321585903 |
| MCI | 0.048458149779735685 |
| Normal | 0.030837004405286344 |Supplemental Figure 2

Supplement: Supplementary file 2 — Figure S2. Clinical Diagnoses of 227 Patients with a Pathologic Diagnosis of Pure AD. More than a third of pathologic AD cases on the Mayo Clinic Brain Bank from 2007 to 2016 were not expected to have AD. (PPTX 52 kb) [file 13024_2019_333_MOESM2_ESM.pptx]
