# Supplementary material for: The neuropathological diagnosis of Alzheimer’s disease
Source: Mol Neurodegener. 2019 Aug 2;14:32. doi: 10.1186/s13024-019-0333-5 (PMC6679484; doi:10.1186/s13024-019-0333-5)
Supplement: Supplementary file 3 — Figure S3. Pathologic Diagnoses in 626 Patients with Clinical Diagnosis of AD. The majority of clinical AD cases as observed in the Mayo Clinic Brain Bank from 2007 to 2016 were found to have co-pathologies. (PPTX 63 kb) [file 13024_2019_333_MOESM3_ESM.pptx]

## Slide 1
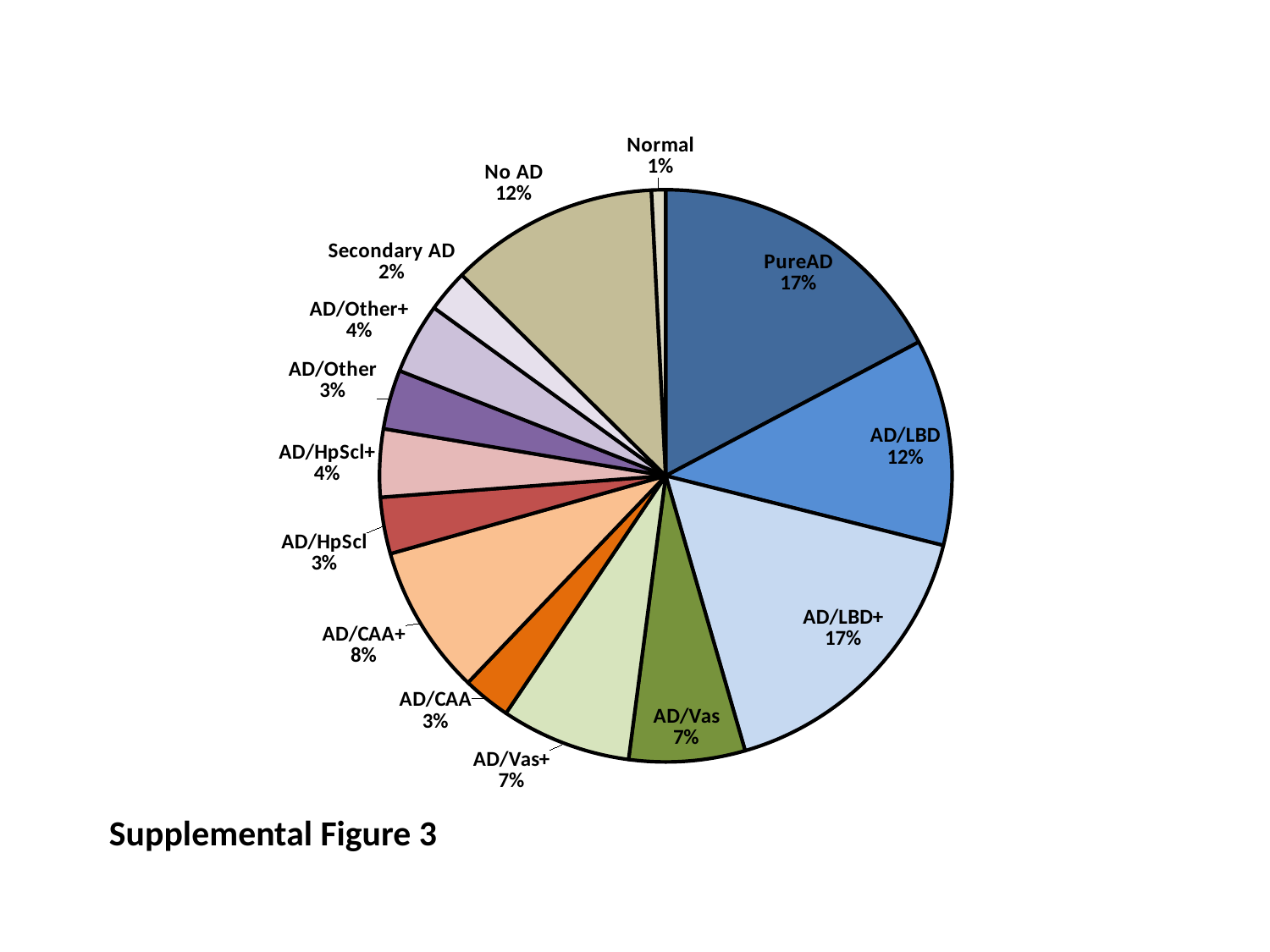

### Chart
| Category | |
|---|---|
| PureAD | 108.0 |
| AD/LBD | 73.0 |
| AD/LBD+ | 104.0 |
| AD/Vas | 41.0 |
| AD/Vas+ | 46.0 |
| AD/CAA | 17.0 |
| AD/CAA+ | 53.0 |
| AD/HpScl | 20.0 |
| AD/HpScl+ | 24.0 |
| AD/Other | 21.0 |
| AD/Other+ | 25.0 |
| Secondary AD | 15.0 |
| No AD | 74.0 |
| Normal | 5.0 |Supplemental Figure 3
